# Update on anterior clinoid process removal in anterior clinoid meningioma surgery: literature review, and a new didactical concept

**DOI:** 10.1007/s00701-025-06742-x

**Published:** 2025-12-23

**Authors:** Kenan I. Arnautovic, Nebojsa Lasica

**Affiliations:** 1 Department of Neurosurgery, The University of Tennessee Health Science Center, Memphis, TN USA; 2 Semmes Murphey Clinic, Memphis, TN USA; 3 Clinic of Neurosurgery, University Clinical Center of Vojvodina, Novi Sad, Serbia; 4 Faculty of Medicine, University of Novi Sad, Novi Sad, Serbia

**Keywords:** Anterior clinoidectomy, Anterior clinoidal meningioma, Skull base surgery, Visual outcome, Optic nerve decompression

## Abstract

**Purpose:**

Anterior clinoidal meningioma (ACM) remains a challenging lesion to treat surgically due to its intricate neurovascular relationships with surrounding anatomy and often presents with ipsilateral visual loss. Anterior clinoidectomy (AC) by skilled skull base surgeons enables early optic nerve (ON) decompression, tumor devascularization, and radical tumor resection. The authors provide an update on ACM surgery, current views on the role of AC and its impact on outcomes in surgical treatment, as well as a new 2 stage 4 by 4 step concept of ON decompression involving AC.

**Methods:**

A systematic review of PubMed and meta-regression of surgically treated ACMs was performed.

**Results:**

In total, 908 patients were analyzed; 415 (45.7%) underwent routine AC (performed in all cases) and 493 (54.3%) underwent selective AC (planned preoperatively). The routine AC cohort showed higher risk for new cranial-nerve (CN) deficits (12.5% vs. 3.0%; *p* < 0.001), vascular complications (6.7% vs. 3.3%; *p* = 0.02), and new focal neurological deficits (5.5% vs. 2.3%; *p* = 0.04). No differences were found in visual outcomes, gross-total resection, mortality, recurrence, or other major complications. Random-effects meta-regression of routine AC showed increased odds of new CN deficit (odds ratio [OR], 3.34; 95% confidence interval [95% CI], 1.51–7.38; *p* = 0.005; heterogeneity [I^2^] = 60.5%) and vascular complication (OR, 2.59; 95% CI, 1.05–6.38; *p* = 0.04; I^2^ = 47.8%), with moderate and substantial heterogeneity among routine AC studies, respectively.

**Conclusions:**

In experienced hands, AC remains an invaluable tool for ACM treatment as it offers more consistent tumor devascularization, prevention of tumor recurrence, optic nerve decompression, and increased working space, which facilitates optimal tumor resection and better long‐term control and functional outcome. We propose a new didactical structured concept of routine AC via 2-stage, 4 by 4 steps to improve the utility of AC and decrease associated operative risks compared to selective AC.

**Supplementary Information:**

The online version contains supplementary material available at 10.1007/s00701-025-06742-x.

## Introduction

Despite advances in skull base neurosurgery, anterior clinoid meningiomas (ACM) remain challenging lesions to treat surgically due to their intricate relationship to major surrounding neural and vascular structures [48]. ACMs most frequently present with progressive visual loss and headache [22]. The reported rate of visual impairment in patients with ACM reaches as high as 60%, possibly due to mechanisms that involve both chronic ischemia and mechanical compression of the optic apparatus (OA) [3, 22]. Although there have been many innovations and advancements in microsurgical techniques over the past few decades—such as AC, opening the falciform ligament, utilizing ultrasonic respirators and high speed drills with cooling irrigation, subarachnoid dissection of blood vessels, to name a few—visual improvement remains dissatisfactory with a pooled rate of 48% reported by a recent study [22].

When compared to meningiomas located more medially (e.g., planum sphenoidale, tuberculum/diaphragma sellae meningiomas), visual outcomes in ACM are reported to be worse [21, 38]. The interference of the tumor with the vascular supply of the OA, along with the associated surgical manipulation of optic nerves (ONs) under pressure during tumor resection, significantly increases the risk of injury and visual deterioration [22, 51]. Currently, the removal of the anterior clinoid process (ACP) is considered integral by many authors, including the pioneering work done by Dolenc and Almefty, as it provides early tumor devascularization, identification, decompression, and decreased tension of the ON, increased working space and reduces the probability of inappropriate manipulation of the OA [1, 21, 28–31, 36, 48].

Successful anterior clinoidectomy (AC) may be hindered by anatomical variations of the ACP and adjacent structures, including pneumatization of the ACP and ossification of the adjacent dural ligaments, which results in the formation of the caroticoclinoid foramen (CCF) and interclinoid osseous bridge (IOB). These variations may increase the risks of cerebrospinal fluid (CSF) leak and internal carotid artery (ICA) injury, respectively [37, 39]. Contemporary ACP removal is predominantly performed via extradural anterior clinoidectomy (EAC) with numerous technical variations; however, both intradural anterior clinoidectomy (IAC) and hybrid techniques have also been documented in the literature [6, 39, 50].

Strategies in the surgical management of these complex skull base tumors and their outcomes vary significantly across the medical literature. This paper aims to provide a summary of relevant anatomy, recent updates, and current evidence on the surgical management of ACMs with a particular focus on the surgical techniques employed and their respective outcomes, as well as a new concept of AC and decompressions of OA.

### Anterior clinoid anatomy overview

The ACP is a bony projection of the lesser wing of the sphenoid bone and the lateral wall of the optic canal [13]. It is characterized by a spiked tetrahedron shape and resembles a caltrop with three bony fixation points at the base that attach to the planum sphenoidale medially, the lesser sphenoid wing laterally, and the optic strut inferomedially [13, 25, 28]. The bony anchors described above serve as key surgical landmarks that are drilled and detached to facilitate release of the ACP [3, 30].

Due to its central location at the cranial base, the ACP has important anatomical relationships with adjacent structures. It forms the anterior part of the roof of the cavernous sinus, and its tip is the site of the attachment of dural folds, namely, the anterior petroclinoid and interclinoid folds [13, 41]. The inferomedial surface of the ACP is closely related to the clinoid and ophthalmic segments of the ICA and the ophthalmic artery [13]. The medial surface of the ACP forms the optic canal and is closely related to the canalicular segment of the ON [3]. Familiarity with these anatomical variations is essential during ACP removal to minimize the risk of potential surgical complications.

### Anterior clinoidectomy technique

EAC has been extensively described elsewhere [3, 29, 30, 33, 39] and is briefly summarized here. Following the cranio-orbital skull base approach, the frontobasal dura mater is peeled off the lateral wall of the cavernous sinus from the floor of the anterior and middle skull base in the region of the lesser wing of the sphenoid bone. This maneuver renders the ACP effectively superficial. The ICA is exposed extradurally, providing an early opportunity for temporary clipping if needed. Subsequent ACP removal is centered primarily on thinning it and disconnecting its bony anchors, which is usually performed with a high-speed drill and abundant irrigation; however, alternative non-drill techniques and bone removal with ultrasonic bone curettes have also been described [1, 6, 14, 23, 28, 30]. The authors prefer using a high-speed drill fitted with a 2 mm diamond burr accompanied by copious cooling saline irrigation to minimize heat generation and reduce the risk for thermal injury to the ON. First, the base of the lesser wing, the posterior portions of the orbital roof and lateral wall orbital wall, and the base of the ACP are removed, followed by layered thinning of the ACP body. After peeling the dura overlying the ACP apex, the optic strut is removed using a 1 mm micro-Kerrison and/or Leksell rongeur. The remaining thinned ACP is then carefully mobilized and removed. Video 1 illustrates the steps of EAC removal and bony optic canal unroofing.

**Fig. 1:**
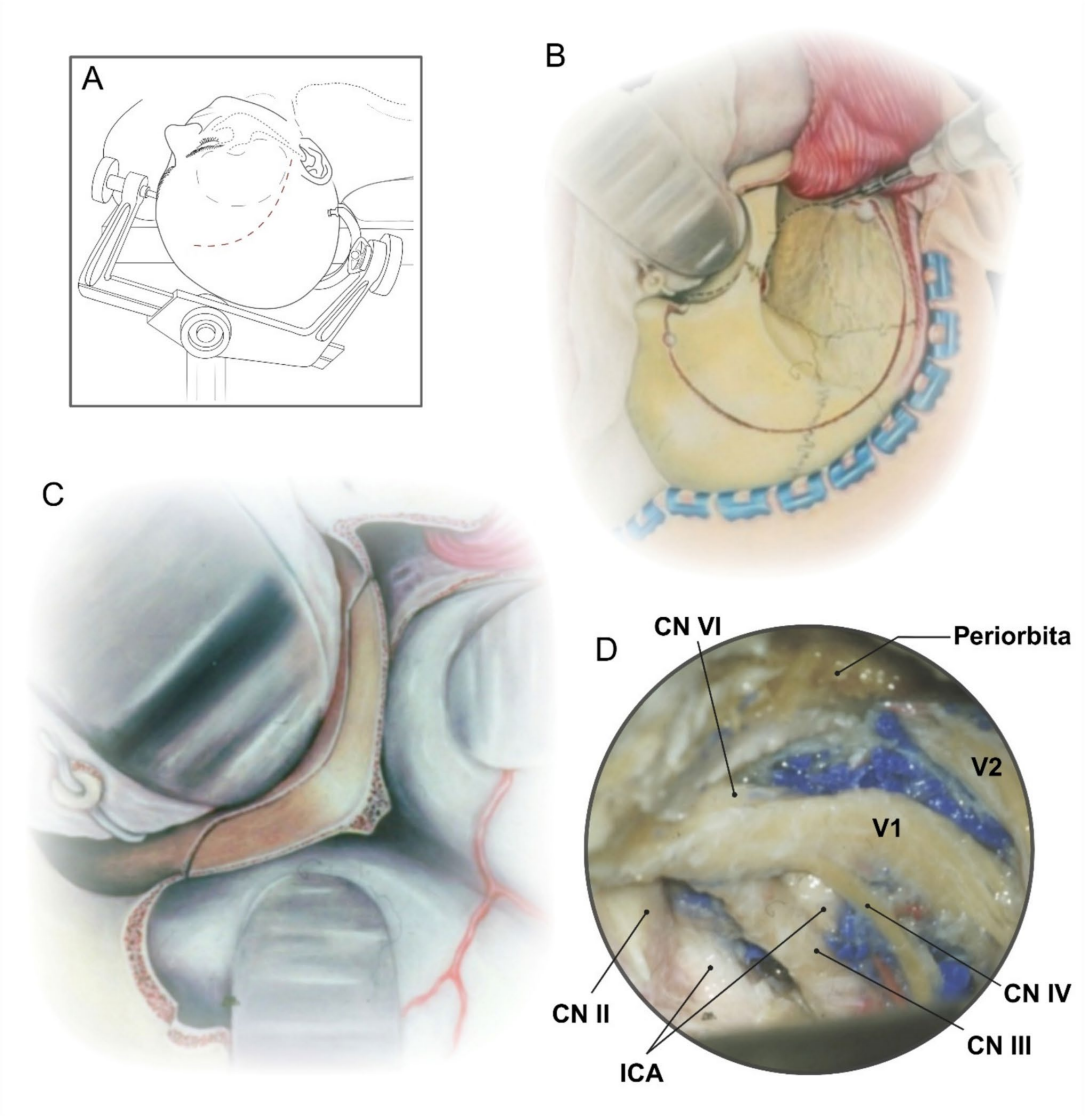
Illustrated stage 1 of the stepwise right sided approach. **A** The patient is positioned supine with the head turned approximately 20°, and a curvilinear skin incision is made from the superior border of the zygomatic arch about 1 cm anterior to the tragus, terminating near the midline behind the hairline. **B** A standard cranio-orbital craniotomy is performed. **C** After release of the meningo-orbital band and dural peeling of the lateral wall of the cavernous sinus, the orbital roof and lateral orbital wall are removed. **D** Exposure reveals cranial nerves III, IV, and the divisions of V (V1–V3) bringing the ACP to the surface

**Fig. 2:**
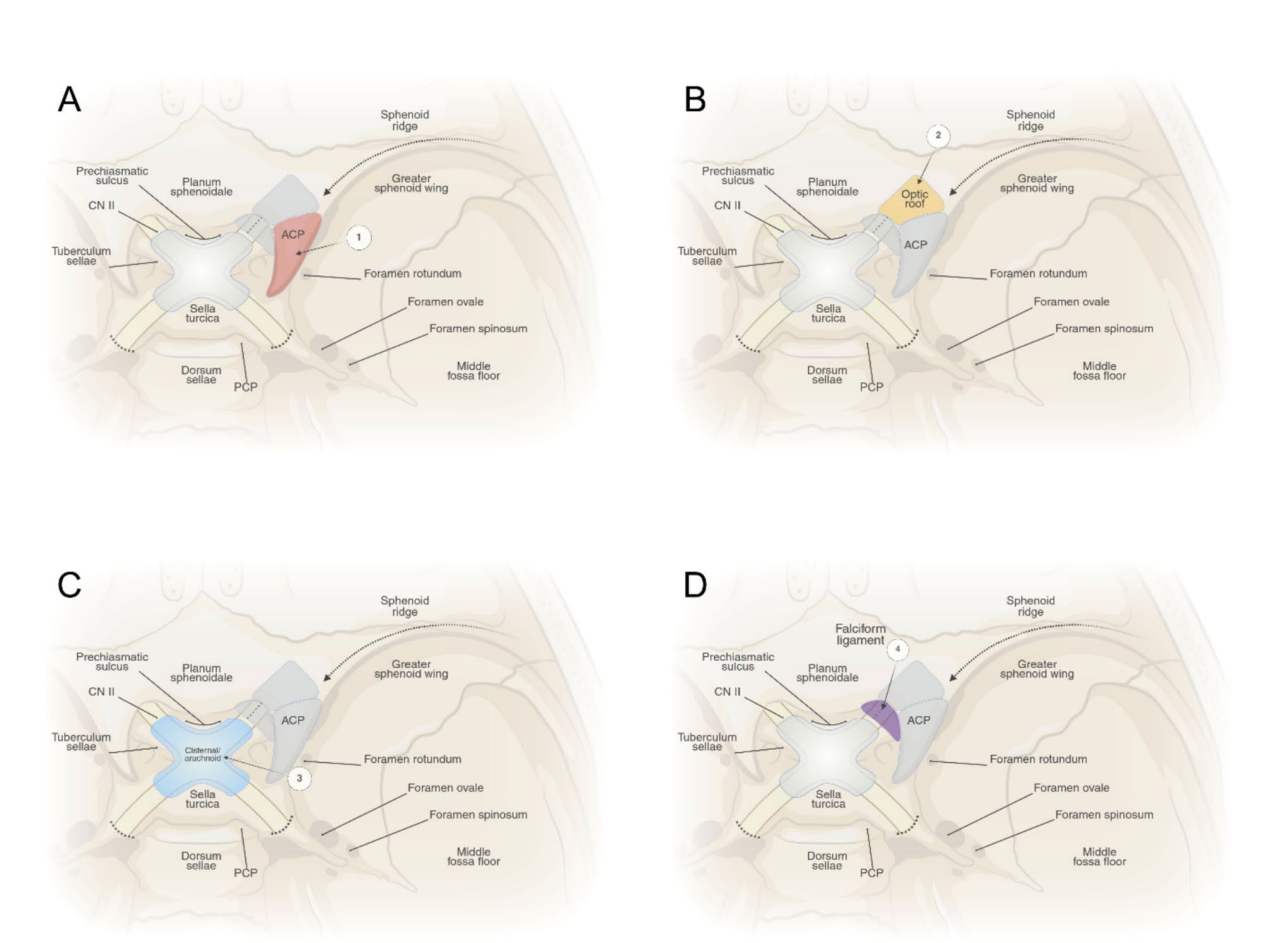
Illustrated stage 2 of the stepwise approach. **A** Extradural clinoidectomy is performed, followed by (**B**) unroofing of the bony optic canal. The basal cisterns are then opened. Thickened arachnoid bands surrounding the ON and ICA are sharply incised (**C**). Final decompression is achieved with the release of the falciform ligament (**D**)

Based on authors’ experience, the EAC is only one part of a two-stage, four-step concept of decompressing the OA. Stage 1 entails the following: (A) patient positioning and elevation of the musculocutaneous flap; (B) cranio-orbital pretemporal approach with opening of the superior orbital fissure; (C) removal of the orbital roof and lateral wall of the orbit to isolate AC; and (D) opening of the lateral dural wall of the cavernous sinus, which brings the AC to the surface (Fig. 1). Stage 2 includes the following: (A) EAC, (B) un-roofing of the optic canal, (C) opening of basal cisterns and releasing of arachnoid bands tethering the OA; and (D) opening of the falciform ligament (Fig. 2). In addition, this technique enables easy proximal access to extradural ICA below the removed AC and the possibility of optional application of temporarily clip, should it become necessary.

IACs, by contrast, are more commonly reported and employed in cerebrovascular surgery, particularly to expose the ophthalmic artery and paraclinoid ICA during aneurysm clip ligation [9]. Proponents of this technique cited the ability to tailor the bony removal under direct visual control of the paraclinoid neurovascular structures. However, this advantage has been disputed, since an EAC affords early exposure and proximal control of ICA [1, 3, 14, 28, 30]. As with EAC, lateral ACP disconnection is performed extradurally through osteotomy of the lesser wing of the sphenoid bone. The remaining steps are performed following the opening of dura. The dura overlying the ACP is incised sharply in a curvilinear or cruciate fashion and reflected. The ACP—and optionally the optic strut and roof of the optic canal— is then drilled, typically with a high-speed diamond burr under copious irrigation, or with ultrasonic bone curettes [2, 9].

In theory, both the EAC and IAC have been described as having distinctive advantages and trade-offs [50]. With EAC, the intact dura protects adjacent neurovascular structures during osteotomy and helps prevent bone dust from entering the intradural space. However, it is reported to be contraindicated in cases of CCF and IOB, since these variants can complicate safe extradural dissection. By contrast, the IAC can be performed when CCF or IOB are present, offering good visual control of critical neural and vascular elements. Nevertheless, it carries a higher risk of neurovascular injury due to the lack of a dural buffer during drilling—raising the risk of mechanical injury—and the potential for bone dust collection in the subarachnoidal space, which has been implicated in postoperative headache [2, 50]. Finally, Meybody et al. proposed a hybrid intradural/extradural approach, supposedly to combine the benefits of purely IAC or EAC technique while mitigating their respective drawbacks [50].

## Materials and methods

### Inclusion criteria

For this review of the literature, we followed PRISMA (Preferred Reporting Items for Systematic Reviews and Meta-Analyses) guidelines and recommendations [40]. Studies were eligible if they included the following criteria: (1) reported surgically treated ACMs; (2) published through December 2024 without the backward time limit; (3) specified the number of patients undergoing AC within the study cohort; (4) clearly defined indications for performing the AC; and (5) published in English. We defined these criteria to select a relatively homogeneous cohort of patients with clinoidal meningiomas while maximizing the sample size.

### Literature search

We performed a PubMed search using the terms “clinoid meningiomas,” “clinoidal meningiomas,” “clinoid meningioma surgery,” “clinoidal meningioma surgery,” “clinoid meningioma outcomes,” and “clinoidal meningioma outcomes,” yielding 276 records. Titles and abstracts were screened by one author (N.L.) under the supervision of the senior author (K.I.A.), resulting in 44 articles for full-text review. Both N.L. and K.I.A. independently evaluated these articles for relevance and extracted demographic, clinical, radiological, and follow-up data. Any discrepancies were resolved by consensus.

**Fig. 3:**
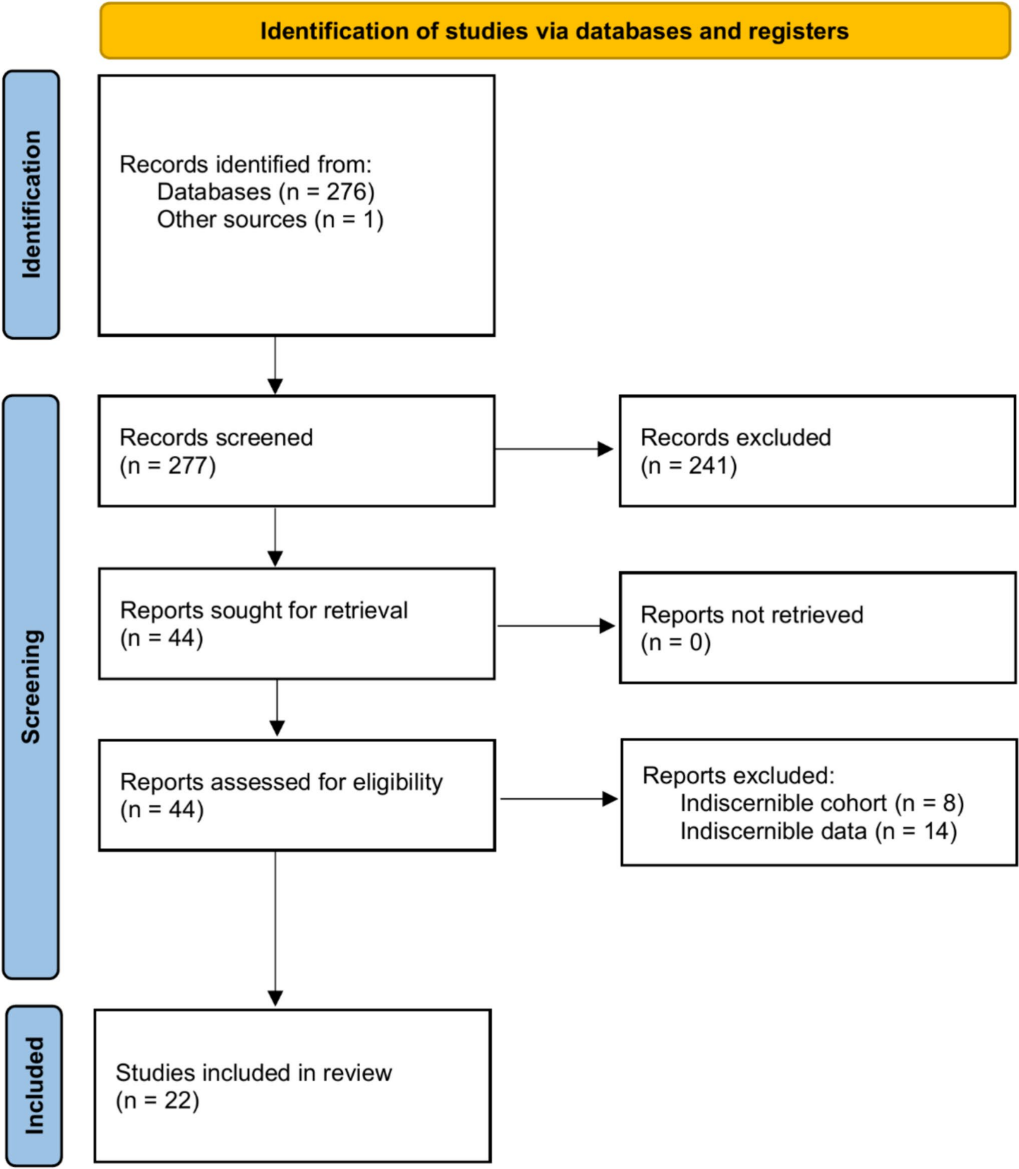
PRISMA 2020 flow diagram depicting study selection, showing the number of records identified, screened, assessed for eligibility, and included or excluded in the systematic review and the subsequent random-effects meta-regression

Of the 44 full-text articles, 22 were excluded due to indistinct cohorts or insufficient data as defined by our inclusion criteria. We also screened the reference lists of the remaining studies for additional eligible reports. The final quantitative analysis comprised 22 studies encompassing 908 patients who underwent surgery for anterior clinoidal meningiomas (Supplemental Data 1) [1, 3, 4, 7, 8, 11, 12, 26, 27, 31, 32, 34, 35, 42–47, 51–53]. Patients were stratified into two cohorts: routine cohort (AC was performed in all cases), and selective cohort (AC was reserved for preoperatively defined indications—e.g., optic canal involvement, clinoid hyperostosis, ICA encasement, etc.). Studies not clearly stating indications for AC were excluded for further analysis (see Fig. 3 for the flowchart outlining the study selection process).

### Statistical analysis

Descriptive analysis was performed initially with categorical variables summarized from all studies and reported using numbers and proportions; continuous variables were shown as means. As part of initial sensibility screening, a between-groups comparison (Routine AC vs. Selective AC) was performed by Fisher’s exact test; outcomes that showed a trend towards statistical significance with *p* < 0.10 were included in the final meta-regression. In that case, we performed a random-effects meta-regression of logit-transformed event proportions, utilizing a continuity correction and estimating between-study variance by the DerSimonian–Laird method. Inverse-variance weighting was used throughout, and surgical strategy (Routine = 1, Selective = 0) was entered as the sole moderator to assess its adjusted effect on log-odds of each outcome. Results were reported via odds ratio (OR) and 95% confidence intervals (95% CIs) and heterogeneity was quantified by I^2^ and τ^2^. All analyses were performed in Python 3.10 using Statsmodels v0.14.0 (Python Software Foundation, Wilmington, Delaware, United States), and forest plots were generated in Matplotlib, a plotting library for Python. A *p*-value of 0.05 or less was considered statistically significant.

## Results

### Study characteristics

Following study inclusion criteria, a combined cohort with a total of 908 patients with ACMs from 22 studies were retrieved from the literature from 1990 to 2024. The size of the cohorts ranged from 10 to 106 patients. Approximately 616 (67.8%) total patients underwent AC, with proportion varying widely in the selective surgical strategy group (range, 10.3–57.7%). According to the available data, mean tumor diameter ranged between 2.1 and 5.1 cm across studies. Invasion of the cavernous sinus was observed in 14–44% of patients, optic canal involvement in 3–80%, and major vessel encasement in 16.7–76.3% cases. Even with considerable reported rates of involvement of adjacent structures, extent of resection was relatively high with gross total resection (GTR) achieved in 60–85% of patients, with recurrence reported in fewer than 15% (range, 0–22.6%).

Visual worsening after ACM surgery across articles ranged from 0–20%, with most studies reporting postoperative deterioration in less than 10% of patients. Other complications were reported variably but followed similar rates across studies. New cranial nerve deficits occurred in 0–35% of patients, vascular complications in 0–29%, and new focal neurological deficits in 0–29%. Cerebrospinal fluid leak, meningitis, seizures, and wound complications were each uncommon and reported to be generally under 5% in most series, with rates ranging 0–6.4%, 0–6.7%, 0–7.6%, and 0–0.9%, respectively. Perioperative mortality was rare (< 3%), ranging from 0–9.5%.

### Quantitative analysis

Of the 908 patients overall, 415 (45.7%) underwent routine AC and 493 (54.3%) underwent selective AC (Table 1). Mean tumor diameter in the Routine AC cohort was 3.6 cm (range, 2.1–5.1), and 3.5 cm (range, 3.0–4.2) in the Selective AC cohort. Within the Selective AC cohort, three studies employed multiple predefined indications for AC, and 8 studies had a single predefined indication for AC. The crude count of ACs performed in the Selective AC cohort was 218 (44.2% of the cohort).

**Table 1:** Results of systematic review showing complications per surgical strategy

Characteristics	Approach		*p* value
	Routine AC	Selective AC	
No. of patients	415 (45.7)	493 (54.3)	-
Mean tumor diameter, cm	3.5	3.6	-
AC performed	415/415 (100.0)	218/493 (44.2)	-
Cavernous sinus invasion	83/342 (24.3)	75/344 (21.8)	0.47
Optic canal involvement	28/129 (21.7)	101/399 (25.3)	0.48
Major vessel encasement	149/282 (52.8)	148/242 (61.2)	0.06
New CN deficit	52/415 (12.5)	15/430 (3.5)	** < 0.001**
Visual worsening	24/335 (7.2)	26/493 (5.3)	0.3
Vascular complication	28/415 (6.7)	14/430 (3.3)	**0.03**
New FND	23/415 (5.5)	10/430 (2.3)	**0.02**
GTR	286/415 (68.9)	326/451 (72.3)	0.3
Mortality	9/415 (2.2)	6/451 (1.3)	0.44
Recurrence	43/394 (10.9)	44/379 (11.6)	0.82
CSF leak	10/394 (2.5)	11/430 (2.6)	0.99
Hydrocephalus	8/415 (1.9)	14/430 (3.3)	0.28
Wound infection	1/415 (0.2)	4/430 (0.9)	0.37
EDH	5/415 (1.2)	2/430 (0.5)	0.28
Meningitis	5/415 (1.2)	3/430 (0.7)	0.5
Seizure	1/415 (0.2)	4/430 (0.9)	0.37

*AC* Anterior clinoidectomy, *CN* Cranial nerve, *CSF* Cerebrospinal fluid, *EDH* Extradural hematoma, *FND* Focal neurological deficit, *GTR* Gross total resection

Values are shown as numbers (%) and mean. Data were summarized across all studies using counts. Some studies did not include all the data

Comparisons across groups were performed using Fisher's exact tests. Boldface type indicates statistically significant *p* value of < 0.05

Among Routine AC patients, cavernous sinus invasion was present in 83 (24.3%), optic canal involvement in 28 (21.7%), and major vessel encasement in 149 (52.8%). In the Selective AC patients, cavernous sinus invasion occurred in 75 (21.8%), optic canal involvement in 101 (25.3%), and major vessel encasement in 148 (61.2%).

Postoperatively, the following differences were found among patients from the Routine AC cohort versus patients from the Selective AC cohort, respectively: new cranial nerve deficits in 52 (12.5%) versus 15 (3.0%); visual worsening in 24 (7.2%) versus 26 (5.3%); vascular complications in 28 (6.7%) versus 14 (3.3%); new focal neurological deficits in 23 (5.5%) versus 10 (2.3%); CSF leaks in 10 (2.5%) versus 11 (2.6%); hydrocephalus in 8 (1.9%) versus 14 (3.3%); wound infections in 1 (0.2%) versus 4 (0.9%); extradural hematomas in 5 (1.2%) versus 2 (0.5%); meningitis in 5 (1.2%) versus 3 (0.7%); and seizures in 1 (0.2%) versus 4 (0.9%). Mortality was 9 (2.2%) in the Routine cohort and 6 (1.3%) in the Selective cohort. GTR was achieved in 286 (68.9%) versus 326 (72.3%), and recurrence occurred in 43 (10.9%) versus 44 (11.6%), respectively.

Fisher’s exact test revealed no statistically significant differences between the cohorts for visual worsening, GTR, mortality, recurrence, CSF leak, hydrocephalus, wound infection, extradural hemorrhage, meningitis, or seizure. It did however identify statistically significant differences between cohorts—compared to patients in the Selective cohort, Routine AC patients had significantly higher risks of new cranial‑nerve deficits (52/415 [12.5%] vs. 15/493 [3.0%]; *p* < 0.001), vascular complications (28/415 [6.7%] vs. 14/493 [2.8%]; *p* = 0.02), and new focal neurological deficits (23/415 [5.5%] vs. 10/493 [2.0%]; *p* = 0.04).

**Fig. 4:**
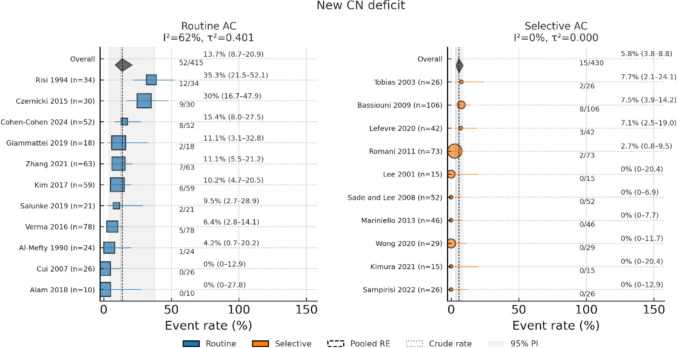
Forest plot of the new cranial nerve deficit determined with a random-effects model

**Fig. 5:**
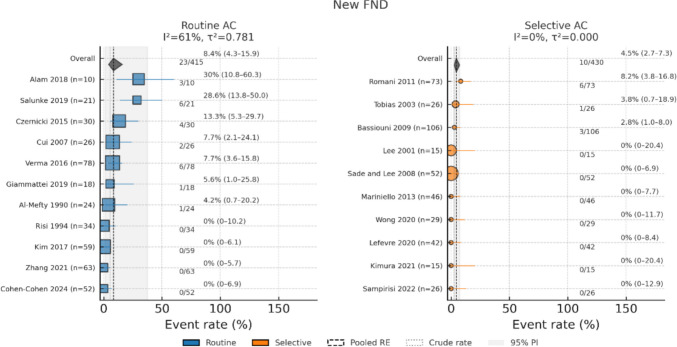
Forest plot of the new focal neurological deficit determined with a random-effects model

**Fig. 6:**
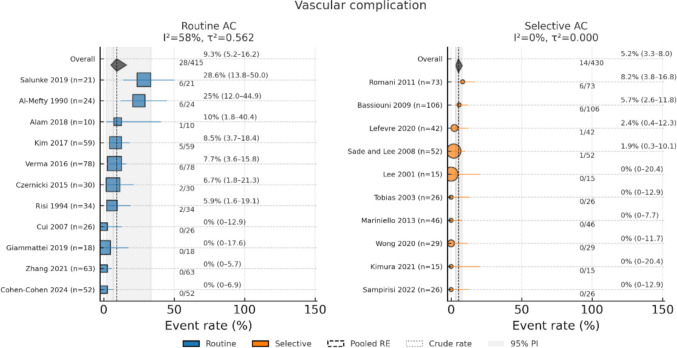
Forest plot of the vascular complication determined with a random-effects model

Results of random-effects meta-regression (Routine = 1 vs. Selective = 0) revealed that the Routine AC was associated with over a threefold increase in odds of new cranial nerve deficit (OR, 3.34; 95% CI, 1.51–7.38; *p* = 0.005; I^2^ = 60.5%; τ^2^ = 0.52) and a 2.6-fold increase in odds of vascular complication (OR, 2.59; 95% CI, 1.05–6.38; *p* = 0.04; I^2^ = 47.8%, τ^2^ = 0.44), with a borderline effect on new focal neurological deficit (OR, 2.89; 95% CI, 0.99–8.43; *p* = 0.05; I^2^ = 53.3%, τ^2^ = 0.66) (Figs. 4, 5 and 6). All three models exhibited moderate or substantial heterogeneity among Routine AC studies.

## Discussion

Removal of the ACP is a key step for both skull base and cerebrovascular neurosurgeons for accessing lesions located in the sellar and parasellar regions [1, 14, 28, 30]. It was first described intradurally by Drake et al. and was truly pioneered and popularized by Dolenc as the extradural removal of the ACP for vascular lesions involving the cavernous sinus in his pioneer contributions [5, 14–19]. Currently, in the context of ACMs, it is considered an integral part of the anterolateral skull base approach due to many advantages that include early tumor devascularization, decreased risk of tumor recurrence (as meningioma frequently invades the ACP and optic canal), decompression of the optic apparatus, decreasing ON tension, increasing optico-carotid space, and reducing the possibility for inadvertent neurovascular injury during the resection [1, 3, 14, 28, 30].

Strategies for AC removal in ACM surgery vary in the medical literature, as do the indications and reported outcomes. Some authors reserve ACP removal for selected cases with specific clinical and radiological features, such as ACP hyperostosis, optic canal invasion, preoperative visual impairment, large and giant size, firm consistency, and ICA encasement [4, 7, 27, 31, 32, 35, 43, 46, 51, 53]. Others, by contrast, perform it routinely in their practice for all ACM cases [1, 8, 11, 12, 22, 26, 28, 30, 34, 42, 45, 47, 52], which is particularly important as ACM frequently invades the ACP and optic canal, and such invasion cannot be reliably predicted on preoperative imaging.

As with many suprasellar meningiomas, one of the most important goals of surgery is visual improvement [20]. A recently published meta-analysis found that studies advocating EAC reported visual improvement rates at the upper end of pooled estimates without a greater risk to the ON [22]. Our analysis similarly showed that routine clinoidectomy was not associated with increased risk of visual deterioration. In the hands of expert surgeons, broader experience with and routine performance of AC carries minimal risk to visual function while potentially reducing tumor recurrence [1, 8, 10, 28, 30].

Results from our meta-regression analysis using surgical strategy as a sole moderator must be interpreted cautiously. While AC clearly offers many advantages in operative treatment of ACMs, such as early decompression and release of pressure and improved vascularization of ON, increased optic carotid space, removal of tumour in the optic canal, and ACP, we found an increased risk of new CN deficit, vascular complication, and new focal nerve deficit associated with routine AC in our literature review. Although the findings may favour a selective AC strategy with respect to complications, heterogeneity among reported rates in studies was moderate (47.8%) for vascular injury and substantial new cranial nerve deficit (60.5%), based on thresholds from Higgins et al. [24]. This variability likely reflects differences in surgeon experience, type of skull base approach used, and annual institutional case volume—these potential sources of inconsistency should be explored in future subgroup analysis or a more comprehensive meta-regression analysis.

We believe that a stepwise extradural bone work extending in the anterior and middle cranial fossae creates a comfortable surgical corridor that allows bringing the AC to the “surface,” permits its safe thinning, facilitates early decompression of the OA, and reduces ON tension. In prior work, we described a stepwise technique for sellar and parasellar lesions—including ACMs—comprising extradural clinoid removal, optic canal unroofing, sharp dissection of thickened arachnoid bands tethering the OA, and incision of the falciform ligament, all important steps performed before safe tumor resection [3, 29, 30]. We hypothesize that this newly introduced didactical concept of 2 stages with 4 by 4 steps decreases the increased risks of routine, compared to selective AC results noted in our literature review, while offering numerous advantages. This finding supported by our experimental work and practice, demonstrated in our series of ACMs and para-sellar tumours, and reported by many other authors [1, 8, 30, 31, 49]. Future prospective studies with larger cohorts and long-term follow-up will be needed to confirm these benefits.

Crude rates of other postoperative complications—including CSF leak, hydrocephalus, surgical site infection, meningitis, extradural hematoma, and seizure—did not show a difference between groups in our analyses. Notably, CSF leak rates were nearly identical among the Routine and Selective AC cohorts (2.5% vs. 2.6%), despite AC often being cited as an independent risk factor for leakage [37]. Similarly, perioperative mortality was low; routine AC did not increase mortality when compared with Selective use (2.2% vs. 1.3%).

### Limitations

This study has several important limitations. First, all data were derived from retrospective studies, which are subject to selection and reporting bias, and no randomized comparison between routine and selective AC exists. Second, substantial between-study heterogeneity (I^2^ up to ~ 60%) likely reflects variability in surgeon experience, case mix, outcome definitions, and operative techniques; the absence of individual patient data precluded adjustment. Third, key variables—such as ACP invasion, cavernous sinus involvement, optic canal tumor extension, and vessel encasement—were underreported or inconsistently defined across studies, resulting in missing data and preventing pooled analyses for some outcomes.

## Conclusion

We reviewed relevant anatomy and relationships of the ACP, technical nuances of AC, and performed a comprehensive systematic review of the literature. The review revealed moderate to substantial heterogeneity among studies of routine AC; therefore, the results must be interpreted cautiously, considering the possible influence of the utilized operative technique, case volume, and surgeon experience with the technique.

While keeping in mind relevant anatomy and clinically important anatomical variations, ACP removal in experienced hands, combined with a cranio-orbital zygomatic pretemporal approach—including removing the roof and lateral wall of orbit, opening the dura of the lateral wall of the cavernous sinus and SOF—expands the surgical corridor for safe ACM resection. This 2-stage with 4 by 4 steps strategy reduces tension on and enables early decompression of the OA, facilitates radical tumor resection, and may be associated with higher improved rates of postoperative visual improvement and lower tumor recurrence, with decreased additional risk or morbidity, and decreasing increased risk discrepancy between routine and selective AC.

## Supplementary Information

Below is the link to the electronic supplementary material.

ESM 1 Supplementary Material 1 (DOCX 24.3 KB)

ESM 2 Supplementary Material 2: Surgical video illustrating key steps and technical nuances of left sided extradural clinoidectomy and additional optic canal unroofing in a patient with left-sided ACM. (MP4 1.07 GB)

## Data Availability

The data that support the findings of this study are available from the corresponding author upon reasonable request.

## Abbreviations

95% CI: 95% Confidence interval
AC: Anterior clinoidectomy
ACM: Anterior clinoidal meningioma
ACP: Anterior clinoid process
CCF: Caroticoclinoid foramen
CN: Cranial nerve
CSF: Cerebrospinal fluid
EAC: Extradural anterior clinoidectomy
EDH: Extradural hematoma
FND: Focal neurological deficits
GTR: Gross total resection
IAC: Intradural anterior clinoidectomy
ICA: Internal carotid artery
IOB: Interclinoid osseous bridge
OA: Optic apparatus
ON: Optic nerve
OR: Odds ratio
