# Supplementary material for: Update on anterior clinoid process removal in anterior clinoid meningioma surgery: literature review, and a new didactical concept
Source: Acta Neurochir (Wien). 2025 Dec 23;167(1):335. doi: 10.1007/s00701-025-06742-x (PMC12743116; doi:10.1007/s00701-025-06742-x)
Supplement: Supplementary file 1 — Supplementary Material 1 (DOCX 24.3 KB) [file 701_2025_6742_MOESM1_ESM.docx]

# **Supplemental Table 1.** Characteristics of ACM patient cohort with tumor characteristics.

| **Authors and year** | **No. of patients** | **AC** | **Mean tumor  diameter, cm** | **CS invasion** | **OC involvement** | **Major vessel encasement** |
| --- | --- | --- | --- | --- | --- | --- |
| Cohen-Cohen et al., 2024 [7] | 52 | 52 (100.0) | 2.1 | 13 (25.0) | 18 (34.6) | 20 (38.5) |
| Chen et al., 2023 [6] | 63 | 33 (52.4) | 4.2 | 9 (14.3) | 22 (34.9) | 44 (69.8) |
| Sampirisi et al., 2022 [41] | 26 | 15 (57.7) | NR | NR | NR | NR |
| Kimura et al., 2021 [22] | 15 | 10 (66.6) | 3.9 | 3 (20.0) | 12 (80.0) | NR |
| Li et al., 2021 [29] | 63 | 63 (100.0) | NR | NR | NR | NR |
| Lefevre et al., 2020 [27] | 42 | 9 (21.4) | NR | NR | NR | NR |
| Wong et al., 2020 [48] | 29 | 3 (10.3) | 3.0 | NR | 7 (24.1) | NR |
| Salunke et al., 2019 [40] | 21 | 21 (100.0) | NR | 1 (4.8) | NR | 13 (61.9) |
| Giammattei et al., 2019 [17] | 18 | 18 (100.0) | 2.7 | 6 (33.3) | 8 (44.4) | 9 (50.0) |
| Shamsul et al., 2018 [42] | 10 | 10 (100.0) | 5.1 | NR | NR | NR |
| Kim et al., 2017 [21] | 59 | 59 (100.0) | 4.0 | 8 (13.6) | 2 (3.4) | 45 (76.3) |
| Verma et al., 2016 [47] | 78 | 78 (100.0) | 4.4 | 21 (26.9) | NR | 43 (55.1) |
| Czernicki et al., 2015 [11] | 30 | 30 (100.0) | 3.7 | 6 (20.0) | NR | 5 (16.7) |
| Mariniello et al., 2013 [30] | 46 | 20 (43.5) | NR | 7 (15.2) | 10 (21.7) | NR |
| Romani et al., 2011 [38] | 73 | 21 (28.7) | 3.2 | 17 (23.3) | 10 (13.7) | 53 (72.6) |
| Bassiouni et al., 2009 [4] | 106 | 23 (22.0) | 3.8 | 31 (29.2) | 16 (15.1) | 51 (48.1) |
| Sade and Lee, 2008 [39] | 52 | 47 (90.4) | 3.4 | NR | 19 (36.5) | NR |
| Cui et al., 2007 [10] | 26 | 26 (100) | 3.5 | 4 (15.4) | NR | NR |
| Tobias et al., 2003 [46] | 26 | 24 (92.3) | 3.7 | 6 (23.1) | NR | NR |
| Lee et al., 2001 [26] | 15 | 13 (86.7) | 3.4 | 2 (13.3) | 5 (33.3) | NR |
| Risi et al., 1994 [37] | 34 | 34 (100.0) | NR | 15 (44.1) | NR | NR |
| Al-Mefty, 1990 [1] | 24 | 24 (100.0) | NR | 9 (37.5) | NR | 14 (58.3) |

AC, anterior clinoidectomy; CS, cavernous sinus; NR, not reported; OC, optic canal.

Values are presented as number of patients (%) or mean.

# **Supplemental Table 2.** Outcomes in ACM patient cohort

| **Authors and year** | **Visual worsening** | **Vascular compli-cation** | **New CN deficit** | **New FND** | **CSF leak** | **Other complications** | **GTR** | **Mortality** | **Recurrence** |
| --- | --- | --- | --- | --- | --- | --- | --- | --- | --- |
| Cohen-Cohen et al., 2024 [7] | 3 (11.0) | 0 | 8 (15.0) | 0 | 1 (2.0) | 1 (2.0) ICH | 36 (69.2) | 0 | 3 (11.0) |
| Chen et al., 2023 [6] | 5 (7.9) | NR | NR | NR | NR | 29 (46.0) NS | 48 (76.2) | 0 | 8 (12.7) |
| Sampirisi et al., 2022 [41] | 0 | 0 | 0 | 0 | 1 (3.8) | 2 (7.6) Seizure | 17 (65.4) | 0 | NR |
| Kimura et al., 2021 [22] | 3 (20.0) | 0 | 0 | 0 | 0 | 0 | 11 (73.3) | 0 | 3 (20.0) |
| Li et al., 2021 [29] | 2 (3.2) | 0 | 7 (11.1) | 0 | 0 | 0 | 50 (79.3) | 0 | 2 (3.2) |
| Lefevre et al., 2020 [27] | 4 (9.5) | 1 (2.4) | 3 (7.2) | 0 | 2 (4.8) | 1 (2.4) Seizure, 2 (4.8) SSI | NR | NR | NR |
| Wong et al., 2020 [48] | 2 (6.9) | 0 | 0 | 0 | 1 (3.4) | 0 | 22 (75.9) | 0 | 6 (17.2) |
| Salunke et al., 2019 [40] | NR | 6 (28.6) | 2 (9.5) | 6 (28.6) | NR | 1 (4.8) Ischemic stroke | 18 (85.7) | 2 (9.5) | NR |
| Giammattei et al., 2019 [17] | 1 (8.3) | 0 | 2 (11.1) | 1 (5.5) | 0 | 2 (11.1) EDH, 1 (5.6) intracranial hypotension | 12 (67.0) | 0 | 0 |
| Shamsul et al., 2018 [42] | 1 (10.0) | 1 (10.0) | 0 | 3 (30.0) | 0 | 0 | 5 (50.0) | 0 | 1 (10.0) |
| Kim et al., 2017 [21] | NR | 5 (8.5) | 6 (10.2) | 0 | 0 | 1 (1.7) SSI, 2 (3.4) HCP | 38 (64.4) | 0 | 11 (18.6) |
| Verma et al., 2016 [47] | 7 (12.1) | 6 (7.7) | 5 (6.4) | 6 (10.2) | 5 (6.4) | 5 (6.4) Meningitis, 1 (1.3) EDH, 3 (3.8) HCP | 52 (66.7) | 2 (2.6) | 16 (20.5) |
| Czernicki et al., 2015 [11] | 3 (16.8) | 2 (6.6) | 9 (30.0) | 4 (13.3) | 0 | 3 (10) Intracranial hypotension, 1 (3.3) brain edema | 19 (63.3) | 2 (6.7) | 4 (13.3) |
| Mariniello et al., 2013 [30] | 1 (3.3) | 0 | 0 | 0 | 0 | 0 | 39 (84.8) | 0 | NR |
| Romani et al., 2011 [38] | 4 (10.3) | 6 (8.1) | 2 (5.4) | 6 (8.2) | 3 (4.1) | 1 (1.4) SSI, 1 (1.4) intracranial hypotension, 8 (10.9) HCP | 57 (78.0) | 3 (4.1) | 3 (4.1) |
| Bassiouni et al., 2009 [4] | 7 (13.5) | 6 (5.6) | 8 (7.7) | 3 (2.8) | 4 (3.8) | 1 (0.9) Wound infection, 2 (1.8) EDH, 3 (2.8) HCP | 62 (58.5) | 2 (1.9) | 24 (22.6) |
| Sade and Lee, 2008 [39] | 0 | 1 (1.9) | 0 | 0 | 0 | 1 (1.9) Meningitis, 1 (1.9) HCP | 37 (71.2) | 1 (1.9) | 0 |
| Cui et al., 2007 [10] | 0 | 0 | 0 | 2 (7.7) | 0 | 2 (7.7) HCP | 16 (61.5) | 0 | 0 |
| Tobias et al., 2003 [46] | 0 | 0 | 2 (7.6) | 1 (3.8) | 0 | 1 (3.8) Meningitis, 1 (3.8) HCP | 20 (77.0) | 0 | 0 |
| Lee et al., 2001 [26] | 0 | 0 | 0 | 0 | 0 | 1 (6.7) Meningitis, 1 (6.7) seizure, 1 (6.7) HCP | 13 (86.7) | 0 | 0 |
| Risi et al., 1994 [37] | 6 (17.7) | 2 (6.0) | 12 (35.2) | 0 | 3 (8.8) | 2 (5.9) EDH, 1 (2.9) seizure, 1 (3.0) HCP | 20 (58.9) | 2 (5.8) | 4 (11.7) |
| Al-Mefty, 1990 [1] | 1 (4.2) | 6 (24.9) | 1 (4.2) | 1 (4.2) | 1 (4.2) | 1 (4.2) HCP | 20 (83.0) | 1 (4.2) | 2 (8.3) |

AC, anterior clinoidectomy; CN, cranial nerve; CSF, cerebrospinal fluid; EDH, epidural hemorrhage; FND, focal neurological deficit; GTR, gross total resection; HCP, hydrocephalus; ICH, intracerebral hemorrhage; NR, not reported; NS, not specified; SSI, surgical site infection.

Values are presented as number of patients (%).
